# Genome-Wide Identification and Characterization of the D-Type Cyclin Gene Family Suggests Its Potential Involvement in Zigzag Stem Formation in Tea Plant (*Camellia sinensis*)

**DOI:** 10.3390/ijms272110021

**Published:** 2026-11-09

**Authors:** Haoran Gao, Saijun Li, Jihua Duan, Yuhong Chen, Yu Lei, Ding Ding, Nianci Xie, Na Tian, Jing Yu

**Affiliations:** 1 College of Horticulture, Hunan Agricultural University, Changsha 410128, China; ghr@stu.hunau.edu.cn; 2 Tea Research Institute, Hunan Academy of Agricultural Sciences, Changsha 410125, China; hncyslsj01@hunaas.cn (S.L.); hncysdjh@hunaas.cn (J.D.); chenyuhong1028@163.com (Y.C.); leiyu0727@163.com (Y.L.); dingding881103@163.com (D.D.); cysxnc@hunaas.cn (N.X.); 3 Yuelushan Laboratory, Changsha 410125, China

**Keywords:** *Camellia sinensis*, D-type cyclins (CYCDs), zigzag-shaped stem, transcriptome analysis, stem morphogenesis, cell cycle regulation

## Abstract

Zigzag-shaped stems are a distinctive architectural trait in tea plants (*Camellia sinensis*), but their underlying molecular mechanisms remain poorly understood. D-type cyclins (CYCDs) regulate cell-cycle progression and proliferation, yet their roles in tea plant architecture remain unexplored. Here, we compared the zigzag-stemmed cultivar *Lianyuanqiqu* with the upright-stemmed cultivar *Zhuyeqi* using phenotypic, anatomical, lignin, and transcriptomic analyses and characterized the *CsCYCD* gene family genome-wide. *Lianyuanqiqu* exhibited locally shortened first internodes, curled leaves, and altered tissue organization, whereas lignin content did not differ significantly between cultivars. Transcriptome analysis identified 4786 differentially expressed genes (DEGs), significantly enriched in plant hormone signal transduction. Genome-wide analysis identified 22 *CsCYCD* genes belonging to six subgroups, with conserved gene structures and promoter cis-regulatory elements associated with hormone responses, growth, and cell cycle regulation. Expression profiling revealed that most differentially expressed *CsCYCD* genes, particularly members of the *CsCYCD3* subgroup, were downregulated in zigzag stems, whereas *CsCYCD1;2* was significantly upregulated. These findings provide the first genome-wide characterization of the *CsCYCD* gene family in tea plants and suggest its potential involvement in zigzag stem formation, offering insights into the molecular regulation of tea plant architecture.

## 1. Introduction

Tea plant (*Camellia sinensis* (L.) Kuntze) is a perennial evergreen woody plant belonging to the genus *Camellia* (Theaceae) and is one of the world’s most important beverage crops. Tea leaves are rich in characteristic secondary metabolites, including tea polyphenols, theanine, and caffeine, which contribute to the distinctive flavor and diverse biological activities of tea. Owing to these unique properties, tea has attracted considerable attention and is now widely cultivated in more than 60 countries [1,2,3,4,5,6]. As a key determinant of plant architecture, stem orientation plays an important role in shaping the overall growth habit and architecture of tea plants, thereby influencing yield formation, field management, and ornamental value [7].

China is the center of origin for the tea plant and harbors the richest tea germplasm diversity worldwide [8,9]. The National Medium and Small Leaf Tea Plant Germplasm Resource Repository (Changsha, China) is a key platform for tea genetic resource conservation in China, preserving numerous accessions including wild resources, landraces, bred cultivars, and elite germplasms. Specialty resources such as albino, purple-leaf, low-caffeine, and zigzag-stem tea plants are valuable materials for germplasm innovation and gene discovery due to their distinct phenotypes and potential utility [10,11]. However, studies on these specialty germplasms have mainly focused on leaf color and quality component formation, whereas the zigzag-stem trait has received little attention, and its genetic basis remains largely unclear [10]. Zigzag-stem tea plants exhibit upward growth with “Z”-shaped stem bending, predominantly at leaf axils (nodes), often accompanied by dwarfism and altered leaf morphology [7,10,11]. This trait confers ornamental value and affects photosynthate transport and mechanical support of aerial parts, making it of considerable research interest [12]. Despite the large repository capacity, zigzag-stem resources are extremely scarce. Representative accessions include “*Qiqu*”, “*Lianyuanqiqu*”, “*Longqu* 1”, and “*Longqu* 2” [11,13,14]. “*Qiqu*” was discovered and vegetatively propagated over 80 years ago, while “*Lianyuanqiqu*” was selected from local populations in Hunan Province; both serve as model materials for studying zigzag-stem formation [7,15]. Research on zigzag-stem tea plants has established a preliminary foundation. Integrated transcriptomic and metabolomic analyses revealed abnormal cell arrangement and morphology in zigzag stems, with differentially expressed genes enriched in phenylpropanoid biosynthesis, flavonoid biosynthesis, and linolenic acid metabolism, alongside changes in genes related to cell expansion, phytohormone regulation, and transcription factors. These findings suggest that alterations in phytohormone-related processes, potentially including auxin transport and gravitropic responses, may be associated with zigzag-stem development [7]. A high-density genetic map localized the controlling quantitative trait locus (QTL) to the 61.2~97.2 Mb interval on chromosome 4 (Chr04), identifying *CsXTH* and *CsCIPK14* as key candidate genes [10]. Other studies have linked zigzag-stem formation to secondary cell wall thickening, lignin accumulation, and abnormal vascular bundle development. By combining bulked segregant analysis sequencing (BSR-seq) with histological sectioning, 1494 differentially expressed genes (DEGs) and 262 candidate single nucleotide polymorphisms (SNPs) were identified. These genes may be involved in phytohormone signaling and biosynthesis, as well as phenylpropanoid-mediated lignin accumulation, potentially contributing to asymmetric vascular bundle development and zigzag-stem formation [11].

Plant growth and development rely on continuous cell division, a process primarily driven by complexes of cyclins and cyclin-dependent kinases (CDKs) [16,17]. The G1-to-S phase transition is a critical checkpoint in this process, with D-type cyclins (CYCDs) serving as key regulators that integrate phytohormonal and metabolic signals, including cytokinins, auxin, and sucrose, to promote cell-cycle progression through the G1/S checkpoint [16,18,19]. CYCDs are highly expressed in actively dividing tissues, including the shoot apical meristem, axillary buds, and vascular cambium [16,18,20]. In Arabidopsis, overexpression of *AtCYCD3;1* promotes leaf cell proliferation, resulting in increased cell number, reduced cell size, and leaf curling, while also sustaining cell division in the absence of exogenous cytokinin [21]. Moreover, loss of *CYCD3* reduces cambial mitotic activity, suppresses radial growth of stems and hypocotyls, and promotes xylem precursor differentiation, demonstrating that *CYCD3* contributes to the coordination of cambial proliferation and xylem differentiation during vascular development and secondary growth [22]. Although the functions of CYCDs have been extensively characterized in herbaceous plants, their roles in perennial woody species remain less well understood. In the hybrid poplar *Populus simonii* Carr. × *Populus nigra* L., *PsnCYCD1;1* is highly expressed in actively dividing tissues, including leaf buds and stems, and localizes predominantly to the nucleus. Its overexpression promotes cell division, enhances cytokinin responsiveness, and affects plant architecture, vascular development, and secondary growth [16,23]. Heterologous expression of *PsnCYCD1;1* in tobacco further induces stem node bending, leaf curling, and reduced epidermal cell size, linking CYCD activity to alterations in plant morphology [20]. Similarly, overexpression of *PtoCYCD3;3* promotes cambial differentiation in *Populus tomentosa*, leaf cell proliferation, and cell expansion in the stem, thereby enhancing vegetative growth. Moreover, its interaction with multiple CDK proteins further supports its role in cell cycle regulation during woody plant development [18]. Mechanical and gravity-related signals can influence plant growth and gene expression, potentially contributing to changes in stem architecture [24]. However, whether these signals are directly associated with CYCD transcriptional variation remains unclear. Evidence from Arabidopsis also suggests that *CYCDs* can influence organ morphology through coordinated regulation of cell proliferation and vascular development. The rippled-leaf phenotype of the activation-tagged mutant rippled leaf is associated with ectopic activation of *CYCD1;2*, whose overexpression produces a similar phenotype accompanied by reduced cell size, increased cell number in the midvein cortex, and a higher proportion of vascular tissue [25]. Collectively, these findings indicate that *CYCDs* link cell-cycle regulation with phytohormonal signaling and vascular development and may thereby contribute to plant architecture and stem morphogenesis. Perturbation of CYCD expression or activity can lead to pronounced morphological alterations, including stem bending and changes in organ architecture, highlighting the potential importance of CYCD-mediated cell proliferation in the establishment of plant architecture.

Although *CYCD* genes have been extensively studied in model plants such as Arabidopsis, their roles in stem and leaf development in tea plants remain poorly understood. To date, no genome-wide study has systematically characterized the *CYCD* gene family in tea plants or explored its potential association with zigzag stem architecture. In this study, the tea germplasm accession *Lianyuanqiqu*, characterized by zigzag-shaped stems and curled leaves, was used as the experimental material, with the upright-stemmed accession *Zhuyeqi* serving as the control. Phenotypic and histological analyses were performed to elucidate the morphological and anatomical features associated with the zigzag-shaped stems and curled leaves. Transcriptome sequencing was subsequently performed to identify DEGs and enriched biological pathways associated with stem development. Particular attention was given to the expression patterns of *CsCYCD* genes potentially involved in cell-cycle regulation. The *CsCYCD* gene family was then systematically characterized through phylogenetic analysis, gene structure analysis, and promoter cis-acting element identification. This study aimed to elucidate the molecular basis of zigzag-shaped stem formation in tea plants with respect to cell-cycle regulation and to provide candidate genes and a theoretical framework for subsequent functional validation and genetic improvement of tea plant architecture.

## 2. Results

### 2.1. Phenotypic Characterization of Upright and Zigzag-Shaped Tea Plant Shoots

**Figure 1:**
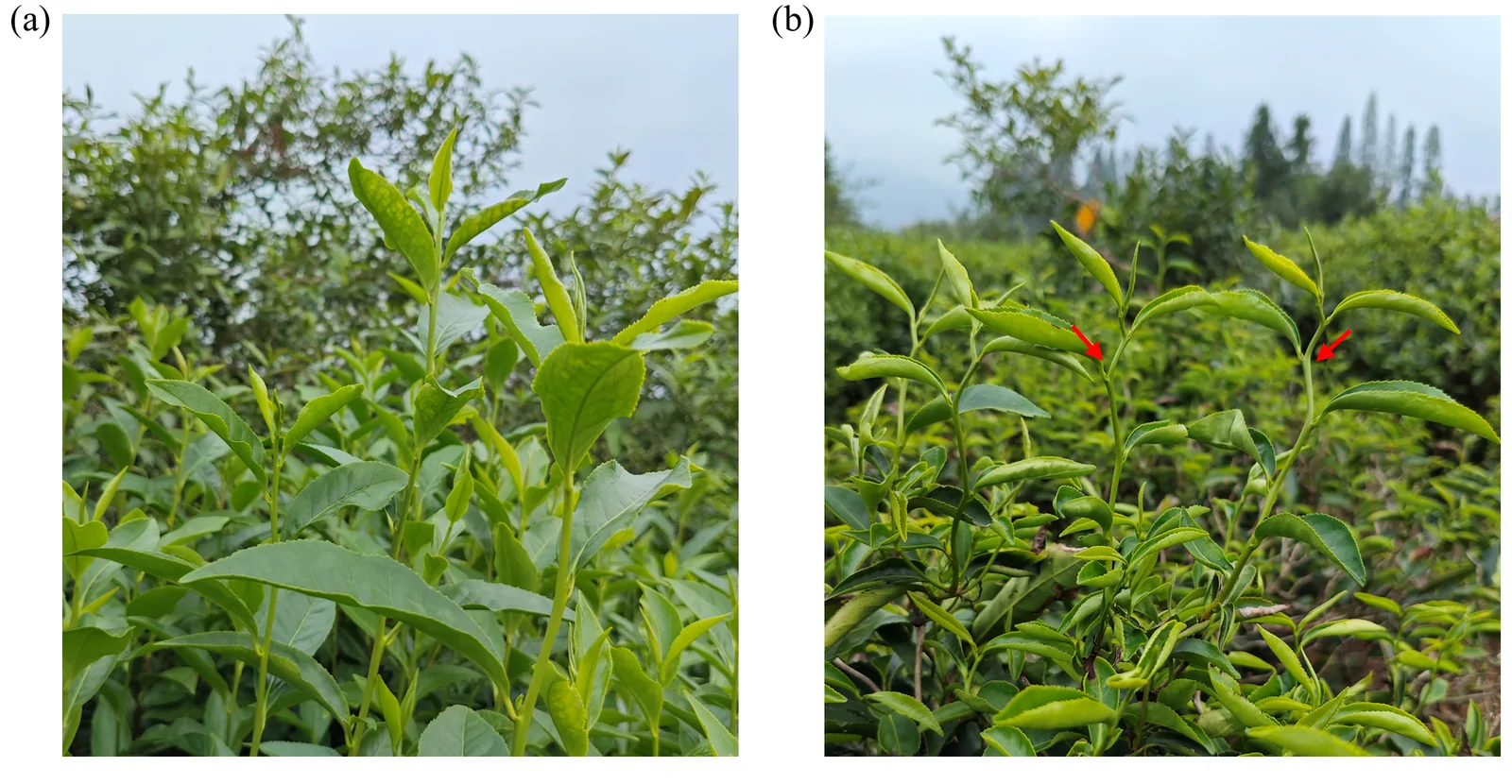
Shoot morphological of *Zhuyeqi* and *Lianyuanqiqu* under natural field conditions. (**a**). *Zhuyeqi* (control), showing a typical upright growth habit with a straight stem. (**b**). *Lianyuanqiqu*, showing a distinct zigzag-shaped stem architecture. Red arrows indicate the bending regions along the stem.

Under natural growth conditions, tea shoots typically display an upright habit, whereas *Lianyuanqiqu* exhibited a distinctly zigzag-shaped architecture. As shown in Figure 1, stem bending began near the apical bud, and curvature directions varied among adjacent internodes, generating a continuously twisted stem. In addition, leaf margins curled inward, restricting lamina expansion. By contrast, the control cultivar *Zhuyeqi* maintained a typical upright habit, with a straight stem and alternately arranged leaves along the shoot axis.

**Figure 2:**
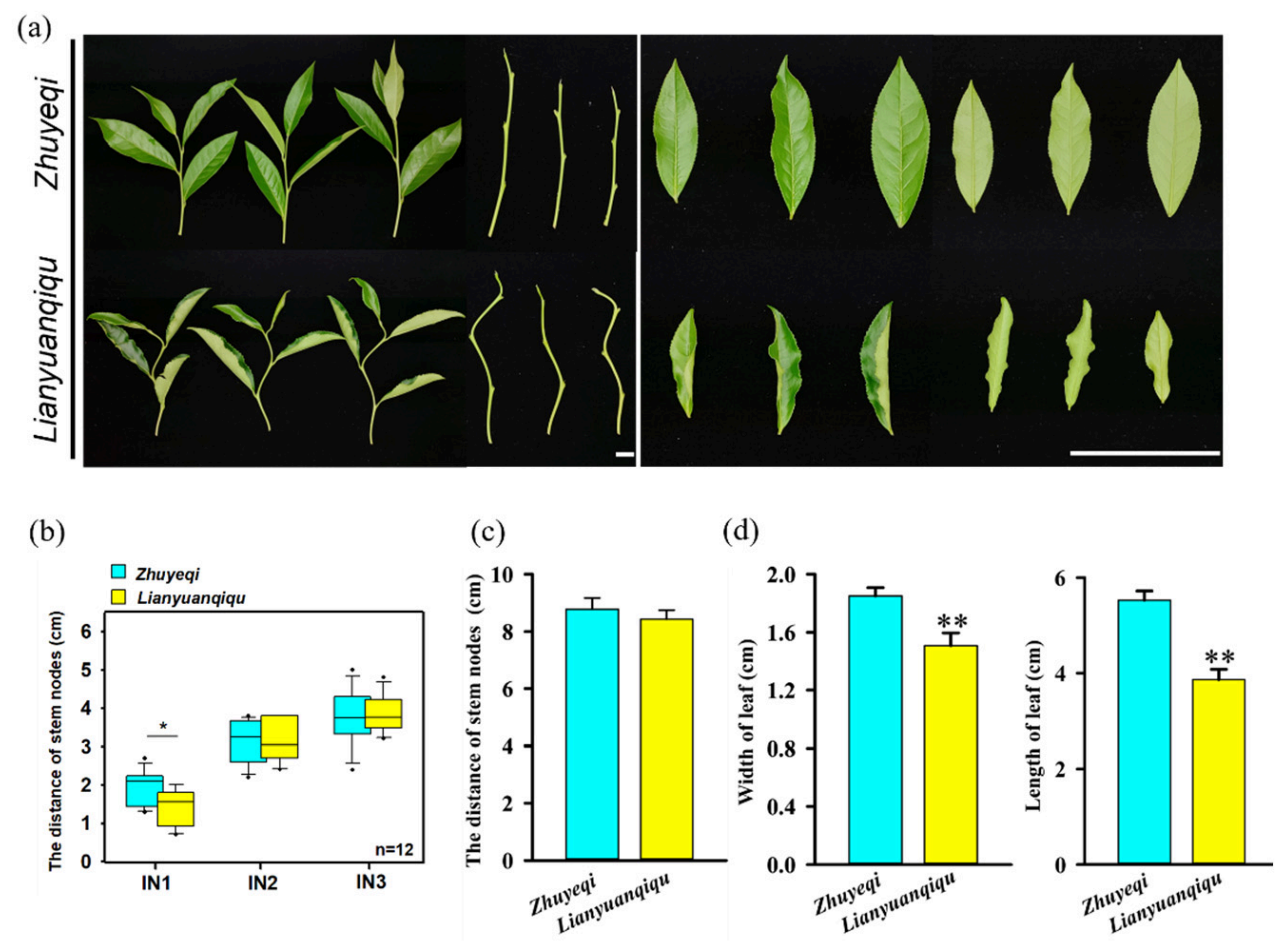
Phenotypic characterization of the *Zhuyeqi* and *Lianyuanqiqu*. (**a**). Phenotype of the stem of upright and zigzag individuals; Bars = 10 cm. (**b**). Internode length of shoots in *Zhuyeqi* and *Lianyuanqiqu*. (**c**). Total length of the three measured internodes. (**d**). Width and length of the second leaf from the shoot apex. n = 12 biological replicates; IN (internode) add a number represents the number of stem internodes; black dots indicate outliers; asterisks represent significant difference by Student’s *t*-test: * *p* < 0.05, ** *p* < 0.01.

To compare shoot morphology between the two tea cultivars, individual internodes lengths and leaf dimensions were measured. The first internode (IN1) of *Lianyuanqiqu* was significantly shorter than that of *Zhuyeqi* (Figure 2a,b). However, when the lengths of all measured internodes were summed, no significant difference in total internode length was observed between the two accessions (Figure 2c). In addition, both leaf length and width were significantly smaller in *Lianyuanqiqu* than in *Zhuyeqi* (Figure 2d). These results indicate that the phenotypic differences in *Lianyuanqiqu* shoots are not manifested as an overall limitation of elongation, but rather as altered growth direction, localized internode shortening, and reduced leaf expansion.

### 2.2. Histological Observations of Leaf and Stem Tissues in Lianyuanqiqu and Zhuyeqi

**Figure 3:**
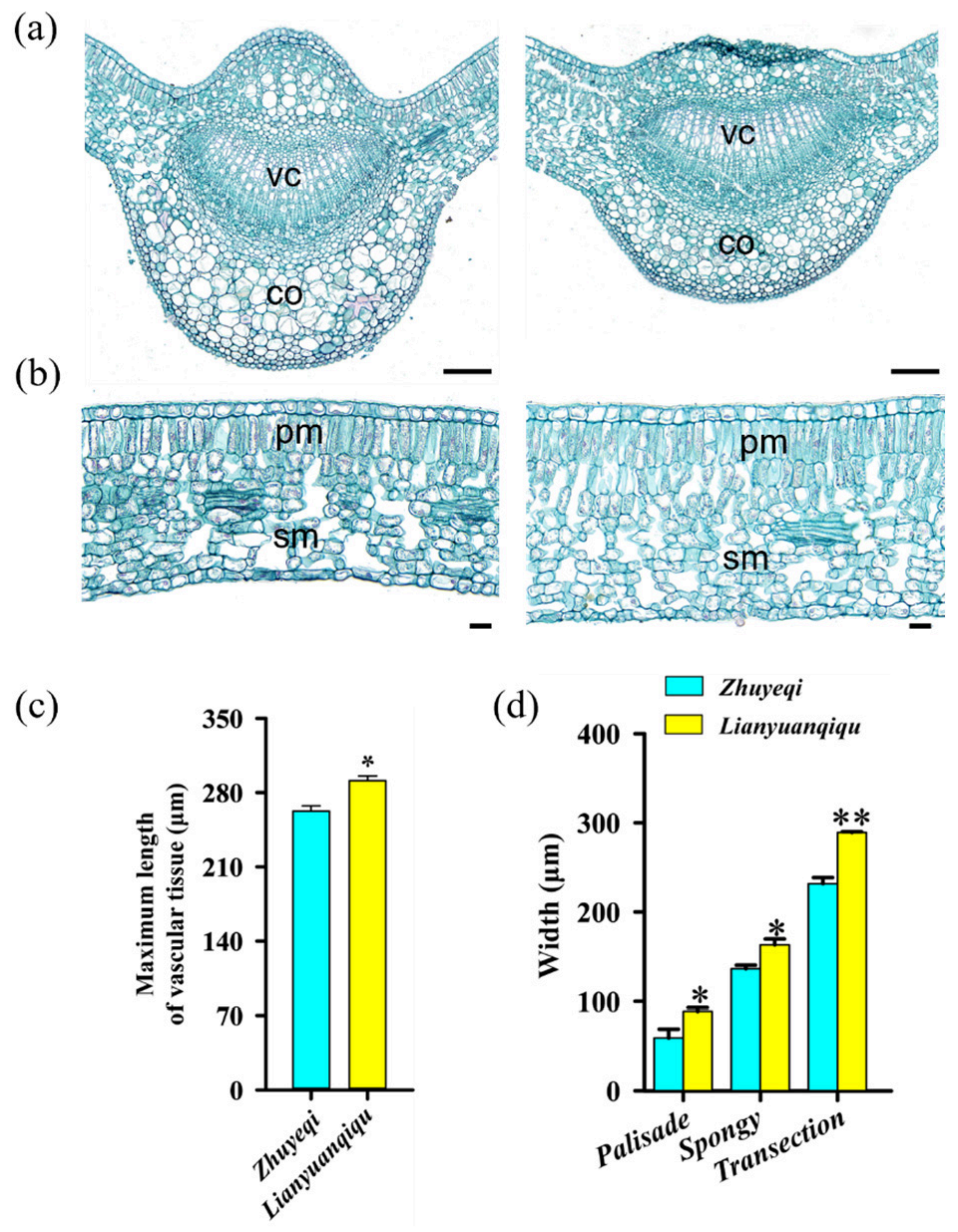
Histological analysis of the leaf of *Zhuyeqi* and *Lianyuanqiqu*. (**a**,**b**) Paraffin sections of the cross-section of *Zhuyeqi* (left) and *Lianyuanqiqu* (right). Bars = 100 μm (**a**); Bars = 20 μm (**b**,**c**) The maximum length of vascular tissue in the leaf blades midrib of two tea cultivars. (**d**) Palisade mesophyll, spongy mesophyll, and transection width of *Zhuyeqi* and *Lianyuanqiqu* leaves. vc, vascular tissue; co, collenchyma; pm, palisade mesophyll; sm, spongy mesophyll. asterisks represent significant difference by Student’s *t*-test: * *p* < 0.05, ** *p* < 0.01.

To investigate the histological basis of leaf curling and zigzag-shaped shoot development in *Lianyuanqiqu*, paraffin sections of leaves and stems from both cultivars were examined. Transverse sections of the leaves revealed clearly defined vascular structures in both cultivars, comprising parenchyma, vascular, and collenchyma tissues (Figure 3a). Compared with *Zhuyeqi*, *Lianyuanqiqu* exhibited significantly greater maximum length of vascular tissue in the leaf midrib (Figure 3c), suggesting differences in midrib vascular organization. However, direct measurements of cambial cell proliferation were not performed. The leaves of *Lianyuanqiqu* were also significantly thicker overall, with thicker palisade and spongy mesophyll layers than those of *Zhuyeqi* (Figure 3b,d). In curled leaves of *Lianyuanqiqu*, the palisade mesophyll appeared more compact and thinner, whereas the spongy mesophyll extended toward the lower epidermis. These findings indicate that leaf curling in *Lianyuanqiqu* is accompanied by structural changes in the vascular bundles and mesophyll, suggesting that altered cell proliferation and tissue differentiation may contribute to this phenotype.

**Figure 4:**
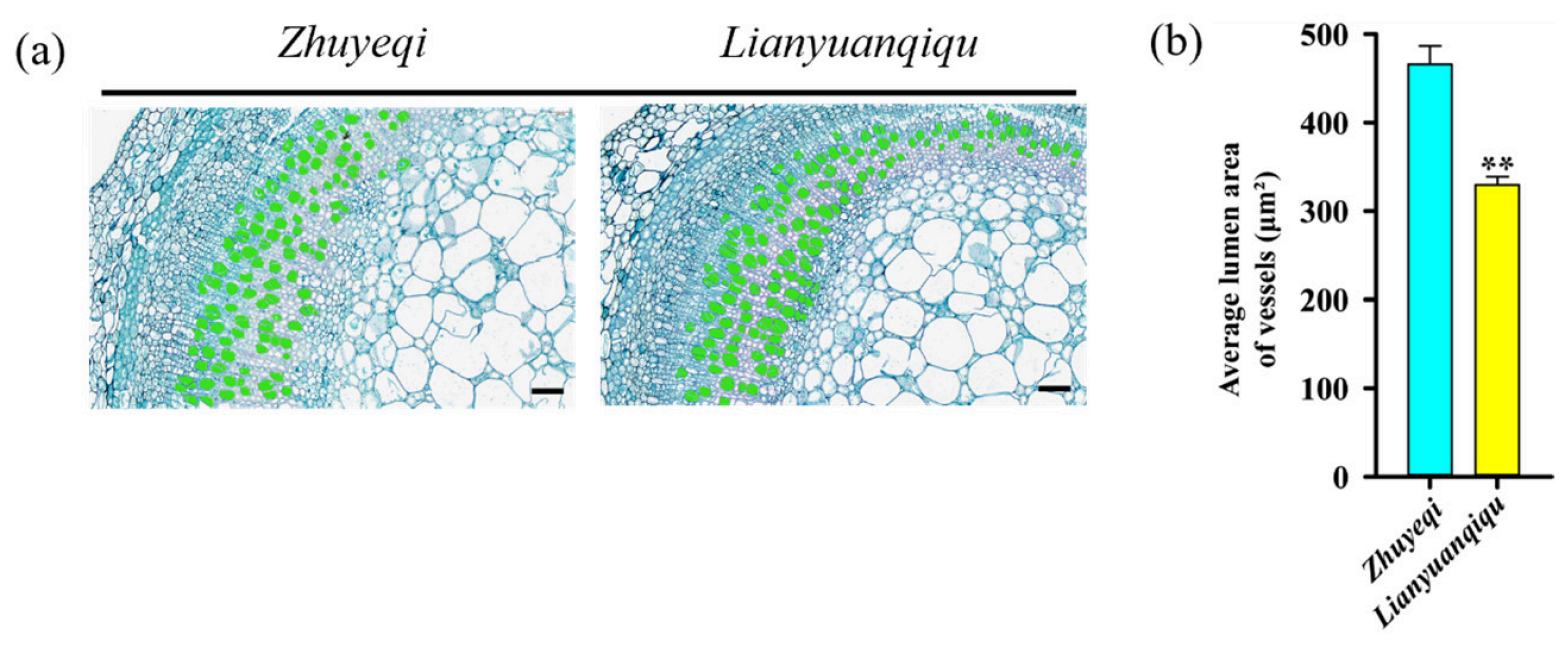
Histological analysis of the stem of *Zhuyeqi* and *Lianyuanqiqu*. (**a**). Stem cross sections of *Zhuyeqi* and *Lianyuanqiqu* showing the size and number of vessel cells in xylem tissues. Vessel cells were marked with green. (Bars = 50 μm) (**b**). Statistical analysis of the mean lumen area of individual vessels. asterisks represent significant difference by Student’s *t*-test: ** *p* < 0.01.

To investigate differences in stem anatomy between the two cultivars, transverse paraffin sections of their stems were examined. Both *Zhuyeqi* and *Lianyuanqiqu* exhibited well-organized stem structures, including the epidermis, phloem, cambium, and xylem. Compared with *Zhuyeqi*, *Lianyuanqiqu* exhibited a significantly smaller xylem vessel lumen area and more densely arranged xylem vessels, as observed in stem cross-sections (Figure 4a,b). These findings indicate differences in xylem vessel morphology and spatial organization between the two cultivars, suggesting that altered xylem development may contribute to zigzag-shaped stem formation.

### 2.3. Lignin Composition Analysis of Stems in Lianyuanqiqu and Zhuyeqi

Histological observations revealed differences in xylem cell number and arrangement between *Lianyuanqiqu* and *Zhuyeqi*. Given the role of lignin as a major component of secondary cell walls in stem lignification and mechanical support, we quantified lignin fractions in bent stem segments of *Lianyuanqiqu* and upright stem segments of *Zhuyeqi*. As shown in Table 1, the insoluble, soluble, and total lignin contents were 28.33 ± 0.24, 2.58 ± 0.02, and 30.91 ± 0.27, respectively, in *Zhuyeqi*, and 28.60 ± 0.13, 2.62 ± 0.05, and 31.22 ± 0.16, respectively, in *Lianyuanqiqu*. None of these differences was statistically significant. Despite differences in stem architecture and xylem organization, the two cultivars exhibited comparable lignin contents, suggesting that differences in lignin accumulation are unlikely to be a major determinant of zigzag-shaped stem formation. Future studies should investigate lignin monomer composition and spatial localization to further elucidate its potential contribution to this phenotype.

### 2.4. Transcriptome Analysis of Zhuyeqi and Lianyuanqiqu Reveals Differentially Expressed Genes and Enriched Pathways

To investigate transcriptional changes associated with zigzag-shaped stem formation, we performed RNA sequencing (RNA-seq) on six samples derived from bent stem segments of *Lianyuanqiqu* and upright stem segments of *Zhuyeqi*. After filtering out adapter sequences, reads containing ambiguous bases (N), and low-quality reads, we obtained 36.46 Gb of clean data, with 38,491,286~43,212,758 clean reads per sample. The Q20 and Q30 values ranged from 98.56% to 98.67% and from 95.49% to 95.88%, respectively, and GC content ranged from 42.78% to 44.64%. These metrics indicated high sequencing quality. The clean reads were then aligned to the *Shuchazao* tea plant reference genome, yielding overall mapping rates of 81.88%~89.11% and unique mapping rates of 77.07%~83.93% (Table 2). These results indicate that the RNA-seq data were of sufficient quality for subsequent differential expression analysis.

**Figure 5:**
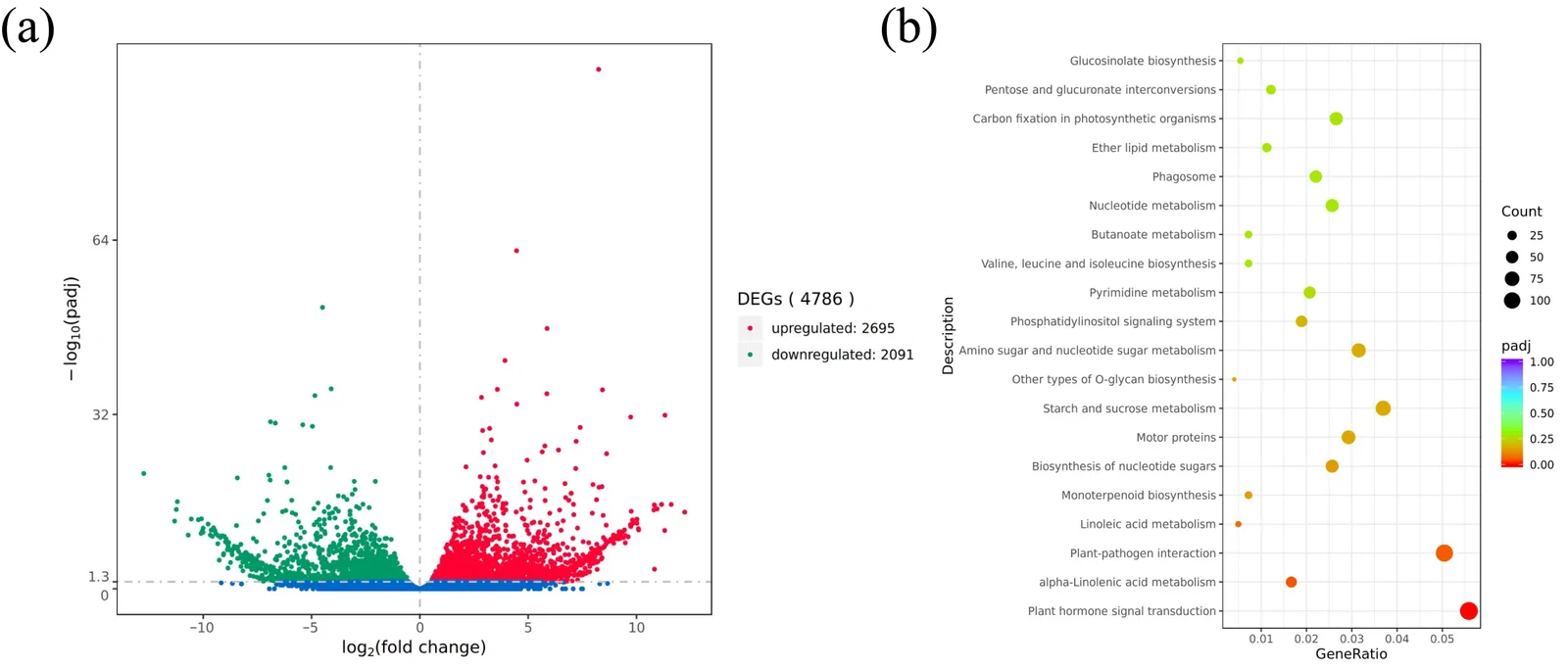
Transcriptome analysis of *Lianyuanqiqu* and *Zhuyeqi*. (**a**). Volcano plot of DEGs in the *Zhuyeqi* versus *Lianyuanqiqu*. Blue dots represent non-differentially expressed genes (non-DEGs). (**b**). Twenty most-enriched KEGG pathways in the *Zhuyeqi* versus *Lianyuanqiqu*.

Comparative transcriptome analysis identified 4786 DEGs between the stem samples of *Lianyuanqiqu* and *Zhuyeqi*, including 2695 upregulated and 2091 downregulated genes in *Lianyuanqiqu* (Figure 5a and Table S1). KEGG enrichment analysis showed that these DEGs were significantly enriched in several pathways, including plant hormone signal transduction (Figure 5b). These results reveal transcriptional differences between the two tea germplasms and provide candidate pathways for further investigation of the molecular basis of zigzag stem development. Abnormal vascular development in curved stems is reportedly driven mainly by hormone content and distribution [16,20,23]. Accordingly, DEGs in this pathway were classified, revealing multiple *CYCD* genes. As CYCD are key regulators of cell cycle progression and cell proliferation, the composition, phylogeny, and structural features of the tea plant *CYCD* gene family were systematically analyzed.

### 2.5. Identification and Characterization of the CsCYCD Family in Tea Plant

Based on the CSS v1.2 reference genome (cv. *Shuchazao*), a total of 22 *CsCYCD* gene family members were identified, of which 21 were derived from existing genome annotations and 1 member, *CsCYCD1;3b*, was obtained through genome-wide TBLASTN searches. These members were classified and uniformly named according to phylogenetic relationships and family member characteristics (Table 3). The 22 *CsCYCD* members were divided into six subgroups—D1, D2/4, D3, D5, D6, and D7—containing 6, 3, 4, 5, 3, and 1 members, respectively, and were distributed across 10 chromosomes (Chr01, Chr02, Chr03, Chr05, Chr06, Chr08, Chr09, Chr10, Chr12, and Chr15). The CsCYCD proteins ranged from 117 to 485 aa in length, with molecular weights of 13.32~55.36 kDa, isoelectric points of 4.78~9.66, and coding sequence lengths of 354~1458 bp.

### 2.6. Phylogenetic Analysis of the CsCYCD Gene Family in Tea Plant

**Figure 6:**
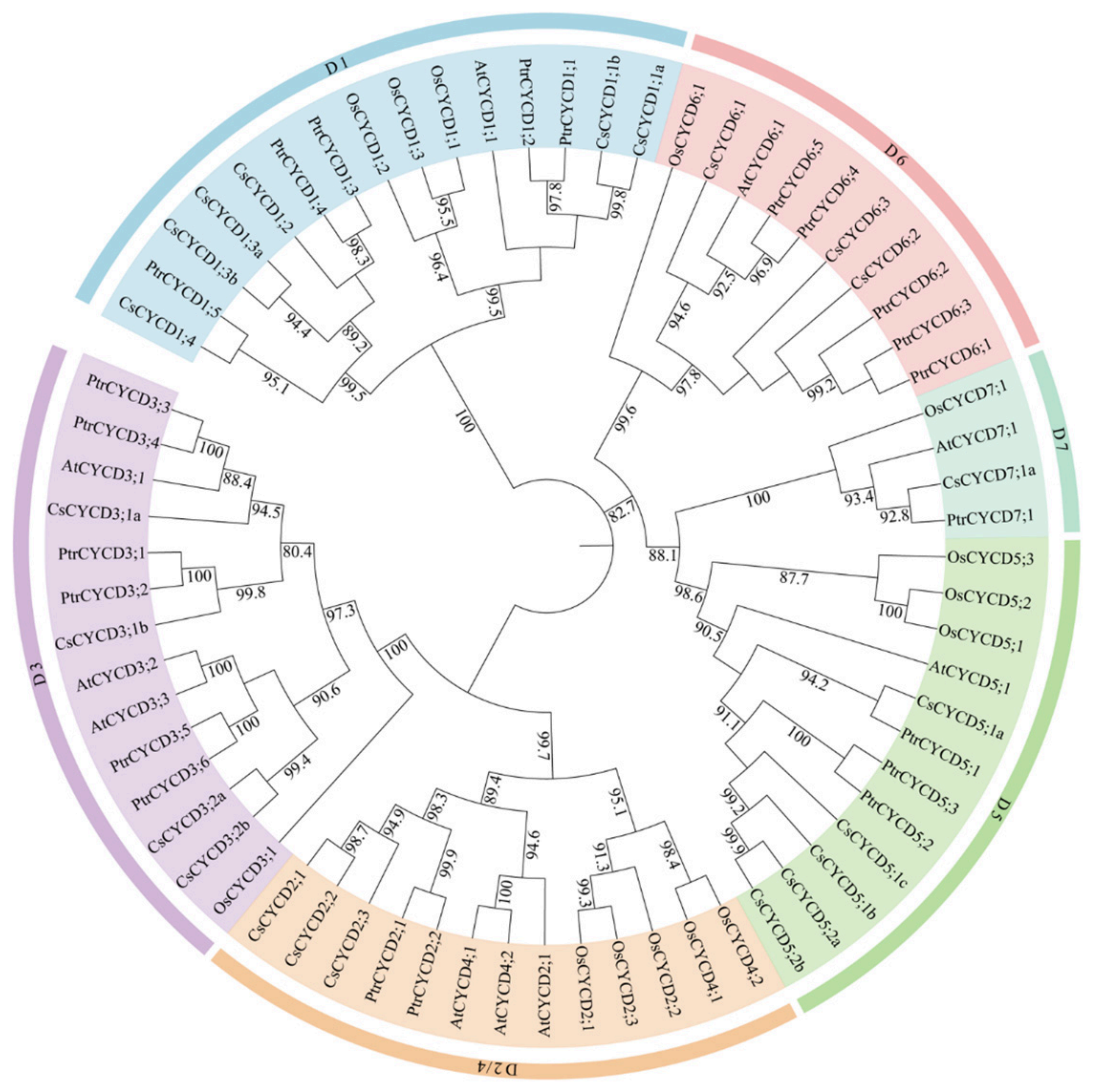
Phylogenetic analysis of CYCD proteins from different plant species. (Cs, *Camellia sinensis*; At, *Arabidopsis thaliana*; Os, *Oryza sativa*; Ptr, *Populus trichocarpa*.) The colored outer sectors represent different CYCD subgroups.

To analyze the phylogenetic relationships among tea plant *CsCYCD*, 22 *CsCYCDs* identified from the *Shuchazao* genome were analyzed phylogenetically with 10 *AtCYCDs* from *Arabidopsis thaliana*, 22 *PtrCYCDs* from *Populus trichocarpa*, and 14 *OsCYCDs* from *Oryza sativa*. Following the *A. thaliana AtCYCD* classification system, 68 CYCD proteins were divided into six subgroups: D1, D2/4, D3, D5, D6, and D7 (Figure 6). Tea plant *CsCYCDs* clustered with CYCD members of corresponding subgroups from other species. For example, CsCYCD3;1a clustered with *A. thaliana* AtCYCD3;1 and poplar PtrCYCD3;3 and PtrCYCD3;4 on the same branch, indicating high sequence similarity among these four proteins. Most tea plant and poplar CYCDs clustered on adjacent branches, indicating a close phylogenetic relationship and high evolutionary conservation of the CYCD family in woody dicots. Moreover, some tea plant CYCDs clustered with *A. thaliana* CYCDs in the same subgroup, suggesting that these genes may retain similar functions in dicots. Compared with dicots, *O. sativa* CYCDs occupied branches distinct from those of tea plant CYCDs, indicating pronounced CYCD family divergence between monocots and dicots. Overall, the tea plant CYCD family exhibits both strong conservation and clear species-specific divergence relative to the *A. thaliana*, *Populus*, and *Oryza* CYCD families, providing a foundation for further functional analysis in tea plant growth and development.

### 2.7. Gene Structure and Conserved Motif Analysis of Tea Plant CsCYCDs

**Figure 7:**
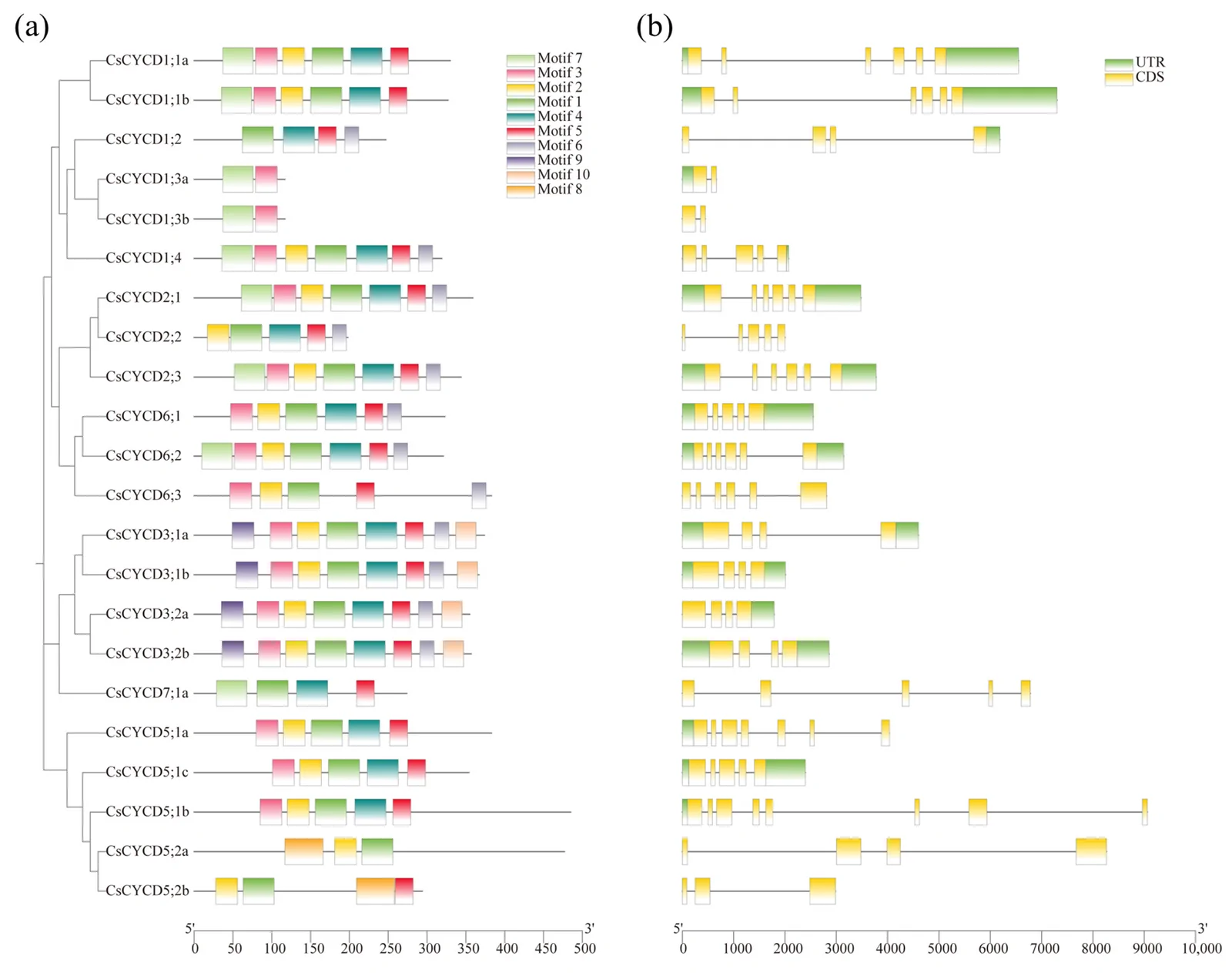
Conserved motif analysis and gene structure of CsCYCD family members in tea plant. (**a**). Conserved motif organization of CsCYCD proteins. The phylogenetic tree is shown on the left, and different colored boxes represent the 10 predicted motifs. (**b**). UTR/CDS structure of *CsCYCD* genes; gray lines represent introns.

Conserved motifs and gene structures were analyzed for 22 CsCYCD proteins to characterize tea plant CsCYCD structural features. MEME analysis identified 10 conserved motifs (motifs 1~10), with 2~8 motifs per protein, each 19~50 amino acids long (Figure 7a and Figure S1). Most CsCYCD proteins contained motifs 1, 2, 3, and 4 in a relatively conserved order, distributed mainly from the N-terminus to the middle region; motif 1 was detected in all 22 members. Closely related CsCYCD members generally shared similar motif composition and arrangement. All four D3 subgroup members, for example, contained eight conserved motifs with largely identical composition and order, and D2/4 and D6 members also showed similar motif composition. In contrast, more distantly related members exhibited motif loss, reduced motif number, or altered arrangement; D1 and D5 members differed markedly in both respects.

Gene structure analysis revealed pronounced exon-intron diversity among the 22 *CsCYCD* genes, yet members within the same phylogenetic branch generally displayed similar structural patterns and conserved motif arrangements. D3 and D6 members, for example, showed similar gene structures consistent with their motif distribution (Figure 7b). This coordinated conservation suggests that gene structure and protein motifs are jointly constrained by family evolution and functional selection. Structurally divergent members also possessed distinctive motif composition; D1 and D5 members showed relatively large structural differences, with *CsCYCD1;3a* and *CsCYCD1;3b* having compact gene structures and *CsCYCD1;1a* and *CsCYCD1;1b* spanning larger regions with longer introns.

### 2.8. Cis-Acting Element Analysis of CsCYCD Gene Promoters in Tea Plant

**Figure 8:**
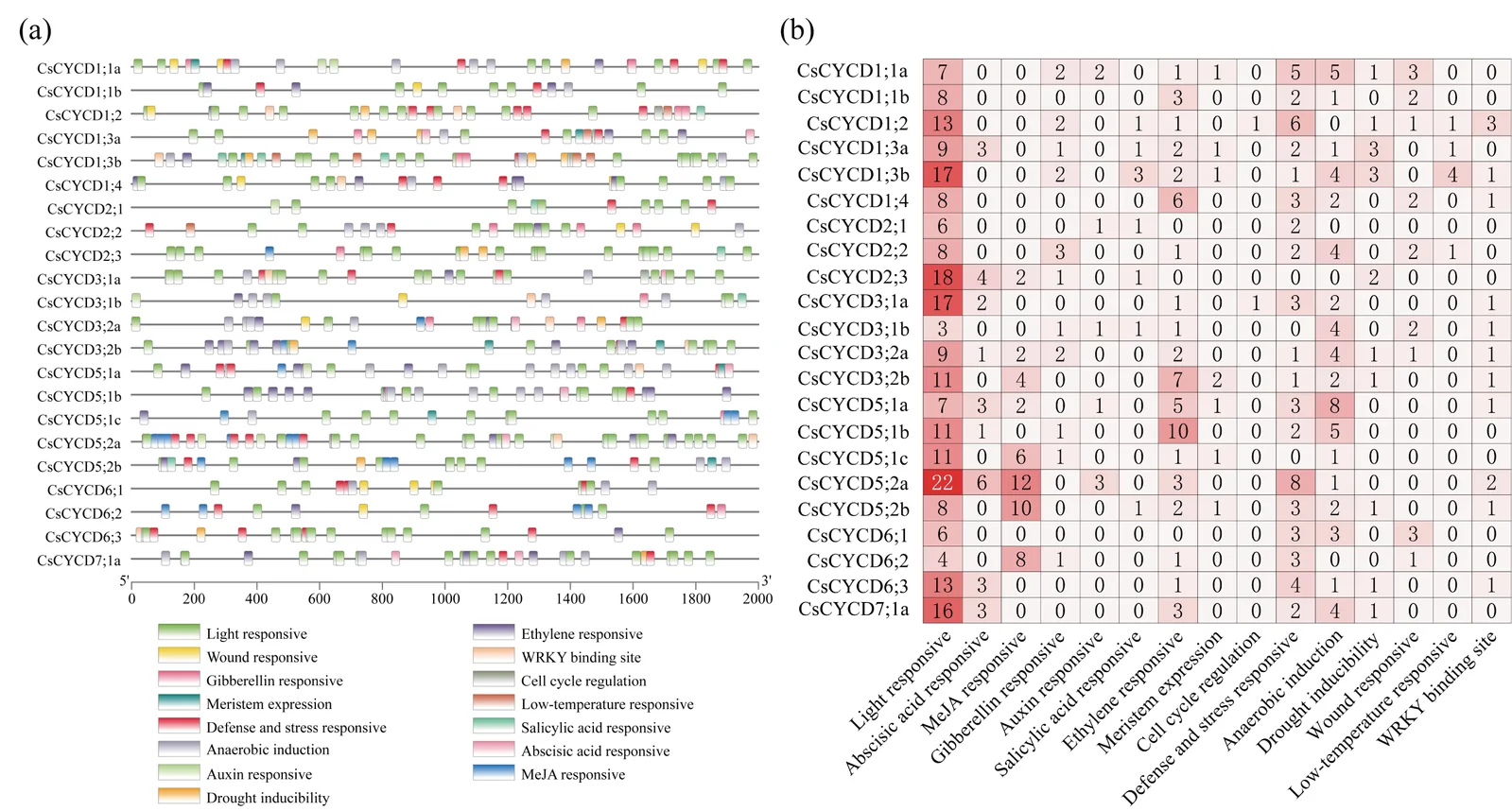
Cis-element analysis of *CsCYCD* gene family in tea plant. (**a**). Types and distribution of cis-elements in the 2000 bp promoter regions of *CsCYCD* genes. Different colored blocks represent different cis-elements. (**b**) Quantitative statistics of cis-elements in the CsCYCD promoter regions. Color intensity indicates the number of elements.

The 2000 bp upstream promoter regions of 22 *CsCYCD* genes were analyzed for cis-acting elements, yielding 15 major classes (Figure 8a) primarily associated with phytohormone response, growth and development, stress response, and light response. Phytohormone-responsive elements were highly diverse, including abscisic acid, MeJA, gibberellin, auxin, salicylic acid, and ethylene response elements, with composition and distribution varying among *CsCYCD* genes. Growth and development-related putative cis-regulatory elements, mainly including meristem expression and cell cycle regulation elements, were present in the promoters of *CsCYCD3;2b*, *CsCYCD3;1a*, and *CsCYCD1;1a*, suggesting their involvement in cell proliferation and organ development. Defense and stress response, anaerobic induction, drought induction, and low-temperature response elements also occurred in multiple CsCYCD promoters. Light-responsive elements were the most prevalent class across CsCYCD promoters, with particularly high numbers in *CsCYCD1;3b*, *CsCYCD2;3*, *CsCYCD3;1a*, and *CsCYCD5;2a* (Figure 8b). Collectively, cis-acting element classes are differentially distributed across CsCYCD promoters, reflecting the regulatory diversity of this family.

### 2.9. Analysis of Differentially Expressed CsCYCD Genes in Tea Plant

**Figure 9:**
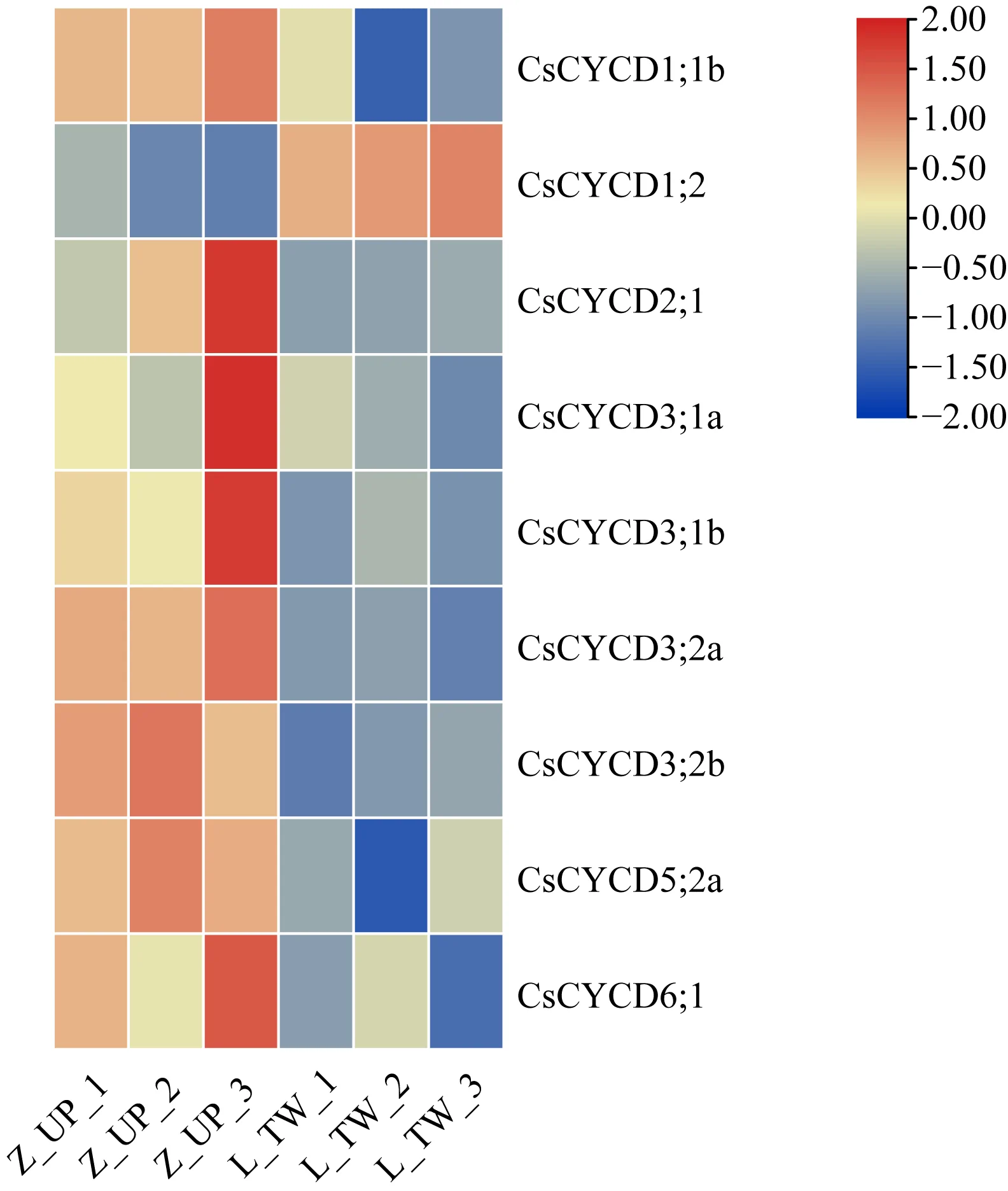
Transcriptome analysis of the transcript abundances of *CsCYCD* genes in stems of *Zhuyeqi* and *Lianyuanqiqu*. Red indicates high transcript abundances and blue indicates low transcript abundances. Z-UP, upright stems of *Zhuyeqi*; L-TW, zigzag-shaped stems of *Lianyuanqiqu*; The numbers 1–3 indicate three biological replicates.

To characterize the expression profiles of *CsCYCD* genes potentially associated with tea plant stem development, nine differentially expressed *CsCYCD* genes were analyzed using transcriptome data from upright stem segments of *Zhuyeqi* (Z-UP) and zigzag stem segments of *Lianyuanqiqu* (L-TW) (Figure 9). The heatmap was generated using row-wise Z-score-normalized log_2_(FPKM+1) values. Eight genes, including *CsCYCD3;1a*, *CsCYCD3;1b*, *CsCYCD3;2a*, *CsCYCD3;2b*, *CsCYCD1;1b*, *CsCYCD5;2a*, *CsCYCD6;1*, and *CsCYCD2;1* were downregulated in L-TW relative to Z-UP, whereas *CsCYCD1;2* was upregulated (Table S2).

**Figure 10:**
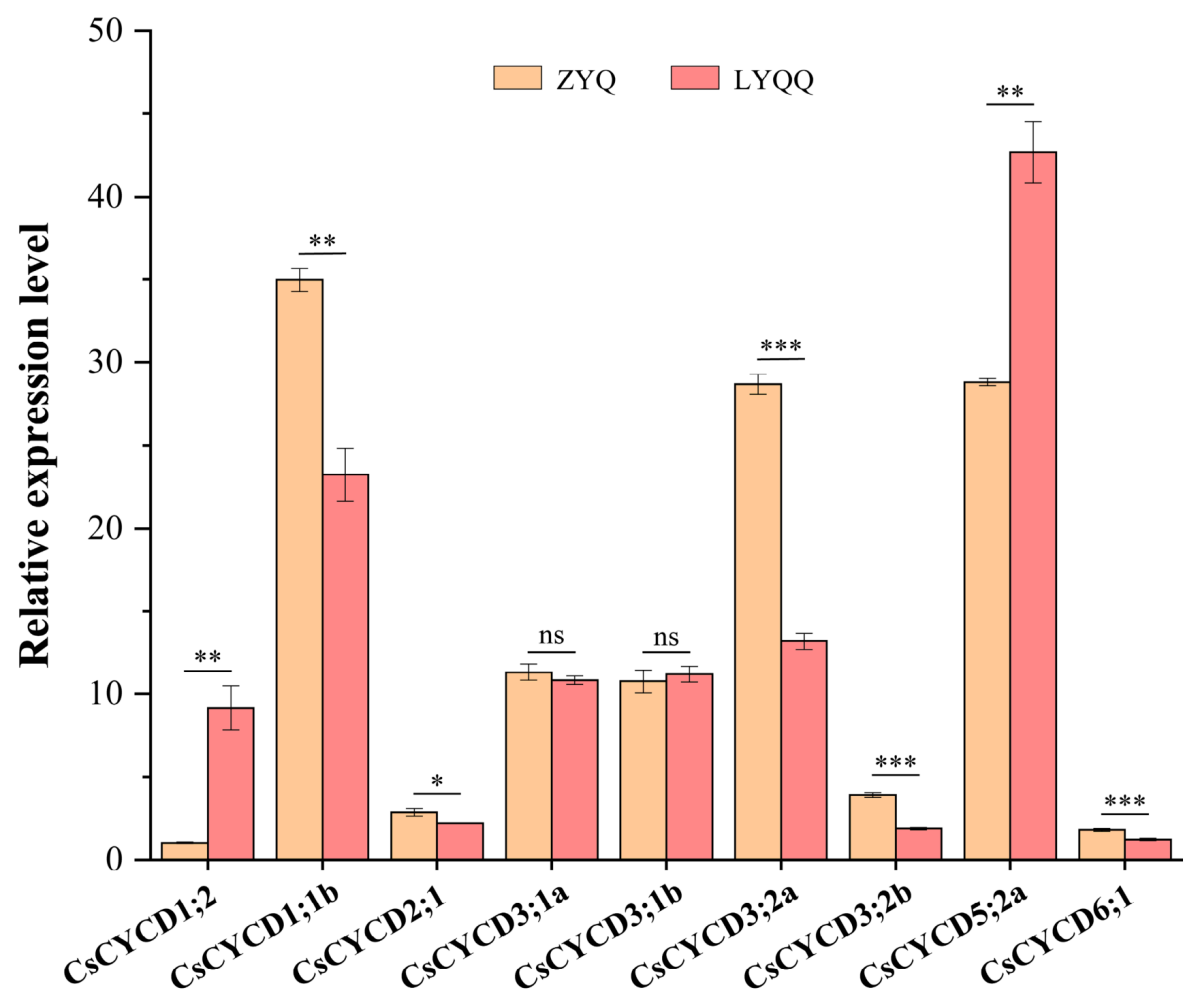
Validation of the relative expression levels of genes using quantitative RT-PCR (qRT-PCR). qRT-PCR results are presented as the means (±SDs) of three technical replicates, utilizing the *CsACTIN* gene as a control. “ns” indicates no significant difference; * indicates a significant difference at the 0.05 level (*p* < 0.05); ** indicates a significant difference at the 0.01 level (*p* < 0.01); and *** indicates a significant difference at the 0.001 level (*p* < 0.001).

To further examine the RNA-seq results, the expression patterns of the nine differentially expressed *CsCYCD* genes were analyzed by qRT-PCR (Figure 10). Six genes (*CsCYCD1;1b*, *CsCYCD1;2*, *CsCYCD2;1*, *CsCYCD3;2a*, *CsCYCD3;2b*, and *CsCYCD6;1*) showed significant expression changes consistent with the RNA-seq results. In contrast, *CsCYCD5;2a* was significantly upregulated in *Lianyuanqiqu* by qRT-PCR, despite being downregulated in the RNA-seq analysis. The expression of *CsCYCD3;1a* remained lower in *Lianyuanqiqu*, whereas that of *CsCYCD3;1b* showed a slight increase; however, neither gene exhibited a statistically significant difference. Overall, the qRT-PCR results partially supported the RNA-seq expression profiles.

## 3. Discussion

Plant stems exhibit diverse architectures, including upright, pendulous, and zigzag forms [12]. Although most are upright, zigzag stems occur in *soybean* [26], *Arabidopsis* [27], *mulberry* [28], and *rapeseed* [29]. Zigzag stems are generally qualitative traits controlled by one or two genes [26,29]. In Arabidopsis, mutations in *AtVTI11* cause the zigzag-stem phenotype in the *sgr4/zig* mutant, which has been associated with impaired vesicle trafficking, altered auxin transport, and abnormal gravitropic responses [27,30,31]. Zigzag stem resources have also been identified in tea plant, including *Qiqu*, *Lianyuanqiqu*, *Longqu* 1, and *Longqu* 2 [11,13,14]. Among these, *Lianyuanqiqu* exhibits continuous zigzag stem curvature, with new shoots curving near the apical bud, variable bending directions between adjacent internodes, inward-curling leaf margins, and a relatively dwarf stature (Figure 1), making it a representative material for studying zigzag stem formation in tea plant. Accordingly, this study used *Lianyuanqiqu* as the experimental material and the upright cultivar *Zhuyeqi* as the control to characterize the anatomical and transcriptional differences associated with the zigzag stem formation.

Histological observations revealed that *Lianyuanqiqu* had significantly reduced xylem vessel lumen area, denser vessel arrangement, greater maximum vascular tissue length in the leaf midrib, and significantly increased leaf, palisade, and spongy mesophyll thickness (Figure 3 and Figure 4), indicating altered vascular and leaf tissue development. These findings corroborate previous reports of abnormal cell arrangement and morphology [7] and support the asymmetric vascular bundle development proposed by BSR-seq analysis [11]. Notably, soluble, insoluble, and total lignin contents did not differ significantly between accessions (Table 1), which contrasts with the previous inference that lignin accumulation drives secondary cell wall thickening, as well as the significant phenylpropanoid biosynthesis enrichment reported previously. Although tissues from the more strongly curved side of *Lianyuanqiqu* stems were sampled, bulk lignin quantification may not capture spatial differences in lignin deposition across the two sides of the stem bend. Thus, zigzag stem formation may involve altered spatial distribution or composition of lignin, such as differences in the lignin monomer S/G ratio, rather than changes in overall lignin content, or programmed remodeling of cell division, differentiation, and arrangement. These findings provide morphological evidence for further investigating the role of cell cycle regulation in zigzag stem formation.

The cell cycle spans the sequence from completion of one division to completion of the next, comprising interphase (G1, S, and G2) and mitotic phase (prophase, metaphase, anaphase, and telophase). Organ development in higher plants relies on tight control of cell division and proliferation throughout the life cycle [20]. D-type cyclins are rate limiting factors for the G1 phase and determine the critical window for cell differentiation [18,20]. Here, 22 *CsCYCDs* were identified from the *Shuchazao* reference genome, divided into six subgroups (D1, D2/4, D3, D5, D6, and D7) and distributed across 10 chromosomes (Table 2). Phylogenetic analysis with 68 CYCD proteins from tea plant, *Arabidopsis*, poplar, and rice (Figure 6) showed that most tea plant *CsCYCDs* clustered with poplar *PtrCYCDs* on adjacent branches but diverged markedly from rice, indicating higher evolutionary conservation of the CYCD family in woody dicots. Members within the same subgroup shared highly consistent conserved motif composition and exon-intron structure (Figure 7); all four D3 members (*CsCYCD3;1a*, *CsCYCD3;1b*, *CsCYCD3;2a*, and *CsCYCD3;2b*) contained eight conserved motifs in identical order. This coordinated conservation suggests that these members are subject to shared evolutionary constraints and retain similar biological functions, providing a basis for functional studies. As rate-limiting factors for the G1/S transition, CYCDs respond to endogenous and exogenous signals such as phytohormones to regulate cell proliferation [16]. Promoter cis-acting element analysis revealed abscisic acid, MeJA, gibberellin, auxin, salicylic acid, and ethylene response elements in multiple CsCYCD promoters, with meristem expression and cell cycle regulation elements additionally present in *CsCYCD3;2b*, *CsCYCD3;1a*, and *CsCYCD1;1a* promoters (Figure 8). This supports phytohormone-mediated CsCYCD regulation, consistent with reports that the poplar *PsnCYCD1;1* promoter is enriched in hormone-responsive elements and participates in stem growth, and that *PtoCYCD3;3* overexpression promotes cambium differentiation and stem cell expansion [18,20]. These findings suggest that *CsCYCDs* may play conserved regulatory roles in secondary growth and stem morphogenesis in woody plants.

Phytohormones are important upstream signals regulating cell-cycle progression and plant development. Auxin and cytokinin, in particular, contribute to vascular cambium activity and can influence CYCD-mediated cell proliferation [32,33]. Thus, the enrichment of plant hormone signal transduction pathways and the differential expression of *CsCYCD* genes indicate differences in hormone-related and cell-cycle-associated transcriptional profiles between the two germplasms. Previous QTL mapping identified a zigzag-stem-associated region on Chr04 of the *Shuchazao* reference genome, with *CsXTH* and *CsCIPK14* proposed as candidate genes [10]. By contrast, the 22 *CsCYCD* genes identified here were distributed across 10 chromosomes, with none on Chr04. This discrepancy may reflect differences between the reference genome assemblies, which limit direct comparisons of genomic positions. A limitation of this study is that *Lianyuanqiqu* and *Zhuyeqi* have distinct genetic backgrounds, making it difficult to distinguish trait-associated transcriptional differences from background variation. Thus, the identified DEGs and enriched pathways should be interpreted as differences between the two germplasms rather than changes specifically associated with zigzag stem formation. Further studies using segregating populations, near-isogenic lines, or multiple independent zigzag-stem accessions are needed to identify trait-associated genes. In addition, RNA-seq samples were collected after the zigzag-stem phenotype had developed; thus, the observed transcriptional differences may include secondary responses to stem bending. Phylogenetic and structural analyses identified the D3 subgroup as a candidate for further investigation in tea plant stem development. Standardized FPKM heatmap analysis (Figure 9) showed that most of the nine differentially expressed *CsCYCDs* were downregulated in curved stem segments of *Lianyuanqiqu*, with all four D3 members (*CsCYCD3;1a*, *CsCYCD3;1b*, *CsCYCD3;2a*, and *CsCYCD3;2b*) and *CsCYCD1;1b* significantly downregulated. CYCD3 is a key rate-limiting factor for cambial cell division and secondary growth; triple-knockout mutant analysis has shown that *AtCYCD3* loss markedly reduces cambial division, suppresses stem and hypocotyl thickening, and promotes premature xylem precursor differentiation [22]. The downregulation of *CsCYCD3* genes in *Lianyuanqiqu* may therefore be associated with altered xylem development, consistent with the observed smaller xylem vessel lumen area and denser vessel arrangement. However, cambial activity was not directly assessed in this study, and the observed anatomical differences cannot be directly attributed to changes in *CsCYCD3* expression. Among the nine differentially expressed *CsCYCDs*, only *CsCYCD1;2* was strongly induced in curved stems. Poplar *CYCD1;2* activation-tagging mutants display pronounced leaf crinkling, with smaller and more numerous cortical parenchyma cells in the midrib and an increased vascular tissue proportion, indicating that altered *CYCD1;2* mediated cell division may affect leaf structure and organ morphology [25]. Given the increased palisade and spongy mesophyll thickness in *Lianyuanqiqu* leaves, the upregulation of *CsCYCD1;2* may be associated with leaf tissue development and warrants further investigation.

## 4. Materials and Methods

### 4.1. Plant Materials

Two tea plant (*Camellia sinensis*) germplasm accessions, *Lianyuanqiqu* and *Zhuyeqi*, were obtained from the National Repository of Tea Plant Germplasm Resources (Changsha, China). Uniformly developed spring shoots were collected for analysis. Internode lengths were measured for the first three stem internodes, numbered sequentially downward from the apical bud. Leaf length and width were measured using the second leaf of each shoot. The third stem node and the second leaf were also collected separately for paraffin sectioning. For wood component analysis, stem segments were collected from the bent regions of *Lianyuanqiqu* shoots, while developmentally matched upright stem segments of *Zhuyeqi* served as controls. For each cultivar, three independent biological replicates were prepared, each pooling stem tissues from at least 10 plants. The remaining fresh samples were immediately frozen in liquid nitrogen and stored at −80 °C for subsequent transcriptome analysis. Three biological replicates were included for each cultivar. Differences between the two cultivars were assessed using an independent-samples Student’s *t*-test, with *p* < 0.05 considered statistically significant.

### 4.2. Histological Analysis

The third stem internode and second leaf from newly developed shoots of *Lianyuanqiqu* and *Zhuyeqi* were collected and immediately fixed in FAA solution. Samples were dehydrated through a graded ethanol series, cleared in xylene, and embedded in paraffin. Stem and leaf sections (16 μm thick) were stained with safranin and fast green, examined using a Precipoint M8 optical microscope (PreciPoint GmbH, Garching, Germany), and imaged with ViewPoint (BETA v1.0.0.0) software. For stem cross-sections, the lumen area of approximately 80 randomly selected intact xylem vessels was measured per biological replicate, and the mean value was used for statistical analysis. For leaf transverse sections, total leaf thickness and the thicknesses of the palisade and spongy mesophyll were measured. Three biological replicates were analyzed for each parameter. Differences between *Lianyuanqiqu* and *Zhuyeqi* were assessed using an independent samples Student’s *t*-test, with *p* < 0.05 considered statistically significant.

### 4.3. Transcriptome Data Analysis

For transcriptome analysis, young shoots of *Lianyuanqiqu* and *Zhuyeqi* at comparable developmental stages were collected. In *Lianyuanqiqu*, an approximately 1.5 cm stem segment centered on the most pronounced bending region was excised, and the outer-side stem tissue at the bending region was collected for RNA-seq. A corresponding 1.5 cm stem segment from *Zhuyeqi* was collected as the control. Three biological replicates were prepared for each cultivar, with each replicate pooled from at least 10 tea plants. Samples were immediately frozen in liquid nitrogen, and total RNA was extracted using a plant RNA extraction kit (Tiangen; DP441, TIANGEN BIOTECH Co., Ltd., Beijing, China). RNA concentration, purity, and integrity were assessed using a NanoDrop 2000 spectrophotometer (Thermo Fisher Scientific, Wilmington, DE, USA) and an Agilent 2100 Bioanalyzer (Agilent Technologies, Santa Clara, CA, USA), respectively. All samples had an RNA integrity number (RIN) ≥ 9.0 and were used for cDNA library construction and Illumina sequencing. Raw reads were filtered to remove adapter sequences, low-quality reads, and reads containing excessive undetermined bases (N). Clean reads were aligned to the chromosome-level reference genome of *Shuchazao* (*Camellia sinensis* var. *sinensis* cv. *Shuchazao*) using HISAT2 (version 2.0.5) [34]. Gene expression levels were quantified as fragments per kilobase of transcript per million mapped reads (FPKM) [35]. DEGs were identified using DESeq2 (version 1.20.0) [36] with adjusted *p*-value (FDR) threshold of 0.05. GO functional annotation and KEGG pathway enrichment analyses were performed for the DEGs, with FDR < 0.05 considered statistically significant. The expression patterns of the nine *CsCYCDs* differentially expressed genes were further examined by qRT-PCR. For heatmap visualization, FPKM values were first transformed using log_2_(FPKM + 1), followed by row-wise Z-score normalization.

### 4.4. Wood Component Analysis

Stem segments were collected from the curved region of newly emerged shoots of *Lianyuanqiqu*, and stem segments from upright shoots of *Zhuyeqi* at the same developmental stage served as controls. After debarking, the samples were soaked in 90% acetone for 2 days and then in 100% acetone for 14 days, with the solvent replaced every 2 days to remove soluble secondary metabolites. The samples were air-dried in a well-ventilated area, finely ground, and analyzed by high-performance liquid chromatography (HPLC) (Agilent Technologies, Santa Clara, CA, USA) for cell-wall component analysis as previously described [37]. Lignin content was expressed as g per 100 g oven-dry wood powder. All measurements were performed with three biological replicates. An independent samples Student’s *t*-test was used to compare lignin traits between the two germplasms, and statistical significance was set at *p* < 0.05.

### 4.5. Identification and Physicochemical Characterization of the CsCYCD Family

CsCYCD family members were identified using the chromosome-scale genome assembly of *Camellia sinensis* var. *sinensis* cv. *Shuchazao* (CSS v1.2) and its corresponding protein, coding sequence (CDS), and GFF3 annotation files [1]. A total of 10, 14, and 22 characterized CYCD proteins from *Arabidopsis thaliana*, *Oryza sativa*, and *Populus trichocarpa*, respectively [17], served as queries for NCBI BLAST+ (version 2.17.0+) and HMM-based searches using PyHMMER (version 0.12.3) against the tea proteome, with an E-value cutoff of 1 × 10^−5^ for both methods [38,39,40]. Candidate proteins were examined for the presence of conserved Cyclin_N (PF00134) and Cyclin_C (PF02984) domains using Pfam (EMBL-European Bioinformatics Institute, Cambridgeshire, UK) (https://www.ebi.ac.uk/interpro/entry/pfam/ (accessed on 25 August 2026)), InterPro (https://www.ebi.ac.uk/interpro/ (accessed on 25 August 2026)), and the Conserved Domain Database (CDD, National Center for Biotechnology Information, Bethesda, MD, USA) (https://www.ncbi.nlm.nih.gov/Structure/cdd/cdd.shtml (accessed on 25 August 2026)), and non-CYCD proteins were removed by phylogenetic analysis with non-D-type cyclin reference sequences [41,42,43]. To identify additional loci, the reference CYCD proteins and candidate CsCYCD proteins were also used in genome-wide NCBI BLAST+ (version 2.17.0+) searches against the CSS v1.2 genome (E-value ≤ 1 × 10^−5^). High-confidence unannotated loci were reannotated with miniprot (version 0.18-r281) and further validated by conserved domain and phylogenetic analyses [44]. Open reading frame (ORF) lengths were obtained from genome annotations, chromosomal positions were corrected using the CSS v1.2 chromosome-level assembly [2], and protein length, molecular weight, and theoretical isoelectric point were calculated with TBtools-II (version 2.467) [45].

### 4.6. Phylogenetic Analysis of the CsCYCD Family

The identified CsCYCD proteins, together with CYCD reference proteins from *Arabidopsis thaliana*, *Oryza sativa*, and *Populus trichocarpa*, were aligned with MAFFT (version 7.526) [39]. A maximum-likelihood phylogenetic tree was inferred in IQ-TREE2 (version 2.4.0) under the best-fit model selected by ModelFinder, with branch support evaluated using 1000 ultrafast bootstrap replicates and 1000 SH-aLRT tests [46]. The tree was visualized using iTOL (version 6; https://itol.embl.de/ (accessed on 26 August 2026)) [47].

### 4.7. Gene Structure and Conserved Motif Analysis of the CsCYCD Family

The exon-intron structures of *CsCYCD* genes were determined from GFF3 annotations and curated gene models. Conserved motifs in CsCYCD proteins were identified using MEME Suite v5.5.9 [48] in Classic mode with the ZOOPS model, limiting the analysis to 10 motifs of 6~50 amino acids and a zero-order background model. The results were visualized with TBtools-II (version 2.467) [45].

### 4.8. Cis-Acting Element Analysis of CsCYCD Promoters

Promoter cis-acting elements of the *CsCYCD* family were identified by extracting the 2000 bp sequence upstream of the translation start site from the reference genome, based on gene annotation and strand orientation. For genes near chromosome ends, the available upstream region was used. Cis-acting elements were predicted with PlantCARE (http://bioinformatics.psb.ugent.be/webtools/plantcare/html/(accessed on 5 September 2026)) [49], functionally classified, and visualized in TBtools-II (version 2.467) [45] by type, number, and distribution.

## 5. Conclusions

In this study, *Lianyuanqiqu* exhibited distinct zigzag stem architecture, localized internode shortening, reduced leaf expansion, and altered tissue organization, whereas lignin content did not differ significantly from that of *Zhuyeqi*. Transcriptome analysis identified 4786 differentially expressed genes, with plant hormone signal transduction significantly enriched. Furthermore, 22 *CsCYCD* genes were identified and classified into six subgroups. Most differentially expressed *CsCYCD* genes were downregulated in *Lianyuanqiqu*, with qRT-PCR partially supporting the RNA-seq results. These findings provide the first systematic characterization of the CsCYCD family in tea plants and suggest its potential association with zigzag stem development. Further functional studies are required to clarify the roles of individual *CsCYCD* genes in regulating tea plant architecture.

## Figures and Tables

**Table 1:** Lignin composition of *Zhuyeqi* and *Lianyuanqiqu*.

Lignin Composition	*Zhuyeqi*	*Lianyuanqiqu*
Insoluble lignin	28.33 ± 0.24	28.60 ± 0.13
Soluble lignin	2.58 ± 0.02	2.62 ± 0.05
Total lignin	30.91 ± 0.27	31.22 ± 0.16

Units: g; values indicate the content per 100 g of oven-dried wood powder; error bars represent the standard error (SE) calculated from three independent biological replicates.

**Table 2:** The statistics of sequencing and mapping data from RNA-seq.

Sample	Clean Reads	Clean Bases	Q20(%)	Q30 (%)	GC (%)	Total Mapping Rate (%)	Unique Mapping Rate (%)
Z_UP_1bio	40574016	6.09G	98.57	95.53	44.51	88.78	83.5
Z_UP_2bio	38491286	5.77G	98.57	95.52	44.64	88.65	83.31
Z_UP_3bio	39154460	5.87G	98.63	95.88	42.78	81.88	77.07
L_TW_1bio	40297818	6.04G	98.57	95.51	44.62	89.11	83.93
L_TW_2bio	43212758	6.48G	98.67	95.82	44.3	88.84	83.56
L_TW_3bio	42085790	6.31G	98.56	95.49	44.39	88.69	83.54

**Table 3:** Basic characteristics of *CsCYCD* family members in tea plant.

Gene Name	Gene ID	Subgroup	Chr	PL (aa)	MW (kDa)	Theoretical pI	CDS Length (bp)
*CsCYCD1;2*	TEA023442	D1	Chr06: Scaffold2990: 214362–220551	247	27.08	5.25	744
*CsCYCD1;3a*	TEA023440	D1	Chr06: 202154728-202155390	117	13.45	6.09	354
*CsCYCD1;3b*	CsCYCDrescue01	D1	Chr06: 202204804-202205253	117	13.32	6.01	354
*CsCYCD1;4*	TEA021007	D1	Chr08: 25208482-25210555	319	36.50	6.14	960
*CsCYCD1;1a*	TEA016706	D1	Chr02: 206671927-206678480	330	36.88	4.78	993
*CsCYCD1;1b*	TEA017808	D1	Chr12: 165788637-165795940	327	36.69	4.91	984
*CsCYCD2;1*	TEA001130	D2/4	Chr10: 75613822-75617301	359	40.06	5.56	1080
*CsCYCD2;2*	TEA017190	D2/4	Chr15: 8171146-8173145	198	22.06	8.50	597
*CsCYCD2;3*	TEA003584	D2/4	Chr01: 41682557-41686333	344	38.38	5.37	1035
*CsCYCD3;1a*	TEA025845	D3	Chr06: 205760221-205764823	374	42.81	4.81	1125
*CsCYCD3;1b*	TEA014197	D3	Chr01: 28125252-28127262	367	41.57	4.83	1104
*CsCYCD3;2a*	TEA004537	D3	Chr03: 226795252-226797041	355	40.51	5.02	1068
*CsCYCD3;2b*	TEA008197	D3	Chr08: 30445410-30448272	357	40.87	5.12	1074
*CsCYCD5;1a*	TEA029991	D5	Chr03: 220939972-220944007	383	42.92	5.86	1152
*CsCYCD5;2a*	TEA005397	D5	Chr10: 178795172-178803438	477	54.16	9.66	1434
*CsCYCD5;2b*	TEA018329	D5	Chr12: 25141523-25144510	294	33.47	6.42	885
*CsCYCD5;1b*	TEA011528	D5	Chr10: 55917884-55926945	485	55.36	7.62	1458
*CsCYCD5;1c*	TEA031243	D5	Chr01: 31755472-31757870	354	40.47	5.13	1065
*CsCYCD7;1a*	TEA008391	D7	Chr12: 47344127-47350907	274	31.87	5.30	825
*CsCYCD6;1*	TEA014131	D6	Chr09: 155417891-155420444	323	36.28	5.16	972
*CsCYCD6;2*	TEA012222	D6	Chr05: 50589081-50592222	321	36.97	5.77	966
*CsCYCD6;3*	TEA001005	D6	Chr09: 185415867-185418678	383	43.28	5.06	1152

Chr, chromosome; PL, protein length; MW, molecular weight; pI, theoretical isoelectric point; ORF, open reading frame. CsCYCDrescue01 represents a *CsCYCD* gene identified through genome-wide TBLASTN searching and subsequent gene model reconstruction.

## Data Availability

All data generated or used during the study appear in the submitted article. The RNA-seq data have been deposited in the National Center for Biotechnology Information Sequence Read Archive under accession numbers PRJNA1552759.

## Supplementary Materials

The following supporting information can be downloaded at: https://www.mdpi.com/article/10.3390/ijms272110021/s1.
