# Supplementary material for: Genome-Wide Identification and Characterization of the D-Type Cyclin Gene Family Suggests Its Potential Involvement in Zigzag Stem Formation in Tea Plant (Camellia sinensis)
Source: Int J Mol Sci. 2026 Nov 9;27(21):10021. doi: 10.3390/ijms272110021 (PMC13763675; doi:10.3390/ijms272110021)
Supplement: Supplementary file 1 [file ijms-27-10021-s001.zip › Figure S1.pdf]

Genome-Wide Identification and Characterization of the D-Type Cyclin Gene Family Suggests Its Potential Involvement in Zigzag Stem Formation in Tea Plant (*Camellia sinensis*)

Supplemental Figures

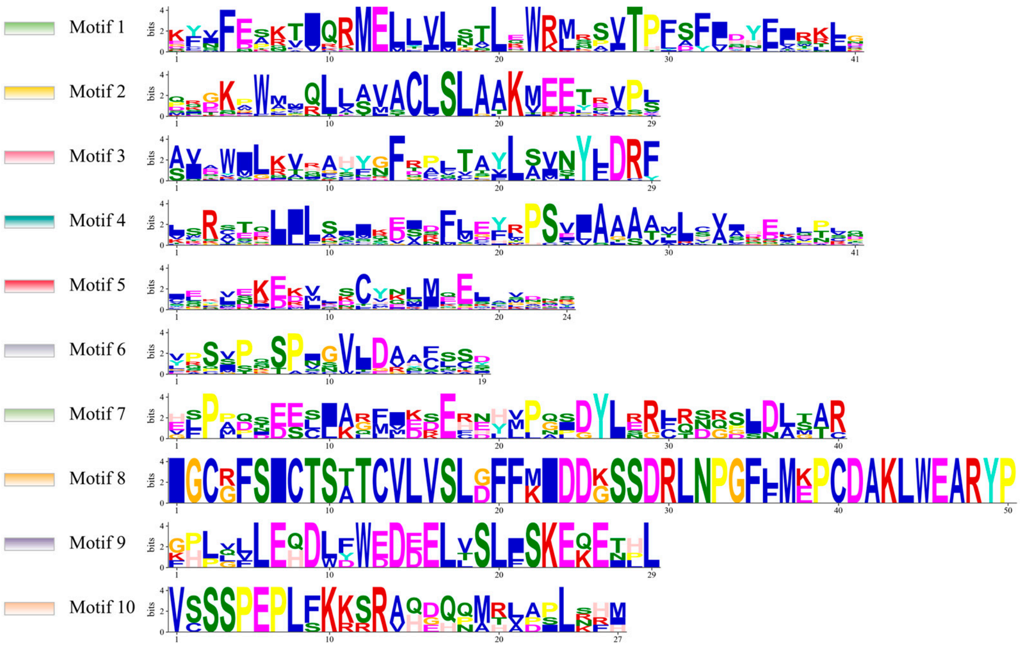

Figure S1. Conserved motif analysis of the CsCYCD family members in tea plant. Different motifs are represented by different colors.
